# An integrated fuzzy logic approach for valuation of power transformer's degree of healthiness or faultiness

**DOI:** 10.1016/j.heliyon.2025.e42789

**Published:** 2025-02-19

**Authors:** J.M. Makacha, Edwell T. Mharakurwa, L.O. Mogaka

**Affiliations:** a Department of Electrical and Electronic Engineering, Dedan Kimathi University of Technology, Private Bag 10143, Nyeri, Kenya; b Department of Electrical and Electronic Engineering, Meru University of Science and Technology, P.O BOX, 972-60200, Meru, Kenya

**Keywords:** Analytic hierarchy process, Degree of healthiness (DOH), Degree of faultiness (DOF), Degradation, Dissolved gas analysis (DGA), Fuzzy logic, Modified combined duval pentagon (MCDP), Power transformer

## Abstract

A substantial number of power transformers that are in use mainly in developing countries are aged and operating beyond their technical design life. This has forced many power utility entities to embrace condition-based maintenance strategies in an effort to prolong assets functionality and reduce equipment failures. To maximize the continuous use of aging power system assets, it is essential to comprehend the variables that pose a threat to the technical and operational lifetime. This paper proposes a model for estimating Degree of Healthiness (DOH) or Faultiness (DOF) of power transformers by synthesizing multiple measured variables, grouped into factors and calculating their corresponding scores. The numerical scores are translated to qualitative assessments through fuzzy logic inference system and thus DOH/DOF derived from the factors is evaluated to give an overall valuation of the in-service power transformer. Furthermore, a nonintrusive degree of polymerization (DP) model based on furans, carbon oxide ratios and methanol as DP pointers is also factored to map the paper insulation condition. Fuzzy rules formulation was centered on variable weighting values established through Analytical Hierarchical Process (AHP) approach. To diagnose the transformer incipient faults, a modified Duval pentagon methodology was employed in interpretation of the Dissolved Gas Analysis (DGA). The accuracy and effectiveness of the established Modified Combined Duval Pentagon (MCDP) technique is high as compared to those of the Pentagon 1 & 2 and combined pentagon methods using the six IEC faults. Results from DOH/DOF evaluation have indicated that DGAF and DPF are more impactful relative to the other factors. Timely knowledge of DOH/DOF of an in-service power transformer can have a great impact in asset managers’ decision making on transformer maintenance and loading management.

## 1 Introduction

Power transformers constitute essential equipment within the power grid, serving the crucial function of converting energy to facilitate transmission and distribution to consumers. They are cost intensive assets of the power transmission network whose operational life cycle should be closely monitored [1,2]. Their safe and stable operation plays a significant role in making sure that the whole power system is reliable, available and runs at an optimal cost. When this equipment fails, clients experience severe disruptions in their energy supplies, substantial financial losses result, and the impact on society is huge [3,4]. An in-service transformer ages with time due to exposure to different changing demands and climatic conditions; consequently, it becomes less resilient to unneeded operational strains and anomalies in the system. Additionally, sometimes the insulating systems encounter harsh electrical, thermal, and chemical operating condition that aids in compromising its technical lifespan. These stressful conditions cause the insulation system to progressively degrade, making the transformer's withstand capability to reduce against system abnormalities such as faults [[5], [6], [7]].

The insulation system in transformers is composed of both mineral oil and solid paper insulation. While DGA provides critical insights into gas formation due to oil degradation, it is also essential to assess the quality of the oil and the condition of the paper insulation. Parameters not limited to the Breakdown Voltage (BDV), Dielectric Dissipation Factor (DDF), and Degree of Polymerization (DP) provide additional crucial insights into the status of the insulation system. To ensure that the transformer is in good condition to operate, assessment of the current condition is crucial. One way to assess the condition from various measurements is to use the condition monitoring approach. To analyze the insulation responses of the solid and liquid dielectrics of the transformers, a number of diagnostic tests have been used, including furan analysis, degree of polymerization (DP), acidity, dielectric dissipation factor (DDF), interfacial tension, flash point, and dissolved gas analysis (DGA) [8]. The gases dissolved in the mineral oil are analyzed by various Dissolved Gas Analysis (DGA) methods such as Near-Infra Red spectroscopy, gas chromatography, photo-acoustic spectroscopy [[9], [10], [11]].

Determining the condition of the power transformer's insulation requires not only analyzing dissolved gases but also evaluating other oil and paper insulation properties. Parameters such as DDF, interfacial tension and DP also provide critical information about the transformer's overall health and durability. The seven major fault gases include the five hydrocarbon gases; hydrogen (H_2_), methane (CH_4_), ethane (C_2_H_6_), ethylene (C_2_H_4_), acetylene (C_2_H_2_) and the two carbon oxides; carbon di-oxide (CO_2)_ and carbon mono-oxide (CO) [12,13]. Various methods have been developed for the analysis of the DGA data to assess the health condition of oil-filled transformers as demonstrated in a flowchart in Ref. [14].

### 1.1 Related literature and gap analysis

Heretofore, innumerable studies have been done on transformer condition valuation, as well as transformer life management. The authors in Ref. [15] suggested a method for calculating the fault degree by using the quantity of energy absorbed or released by a paraffinic oil compound known as eicosane (C_20_H_42_) when it faults and breaks down into hydrocarbon gases; H_2_, C_2_H_2_, CH_4_, C_2_H_4,_ C_2_H_6_ and cellulose breakdown to produce CO, H_2_, and CO_2_. Researchers in Ref. [16] have put forward a similar analogy but utilizing the n-octane (C_8_H_18_) molecule. In both cases they estimated the severity of incipient faults based on the gases evolved when these molecules decompose. Though the method proved effective in determining the extent of insulation damage, it did not determine the extent of damage on other key components of the power transformer as a whole. The exact molecule that produces the dissolved gases are still yet not clear, additionally, whereas the studies focused on thermodynamic approach of the gas formations as a key factor, the influence of other variables such as loading and operational stress influence was not explored.

In reference [5], the authors assessed the Degree of Healthiness (DOH) and Degree of Faultiness (DOF) of transformers by analyzing Dissolved Gas Analysis (DGA) data. They utilized the carbon dioxide to carbon monoxide (CO_2_/CO) ratio along with the Rogers ratios, which include the gas ratios CH_4_/H_2_ (methane to hydrogen), C_2_H_6_/CH_4_ (ethane to methane), and C_2_H_4_/C_2_H_6_ (ethylene to ethane). The evaluation was based on the extent to which these gas ratios deviated from standard reference values, allowing them to quantify the transformers' health and fault conditions. The main challenge with Rogers's ratios is that different gas concentrations would still give the same value, this might lead to wrong interpretation since the gases are generated at different fault energies. Additionally, DGA alone is not a meticulous science and thus suffers limited fault specificity. If the value of the gases' ratio is close to the limit, it provides an incorrect diagnosis or stays ambiguous, thus failing during complex categorization. Moreover, the authors in Ref. [5] only addressed DOH/DOF basing on the incipient faults, the knowledge on overall transformer DOH/DOF addressing the quality of oil, the status of the degree of polymerization and the maintenance history was not considered in the scope of their study.

An approach for determining the health index of power transformers based on sample data acquired from oil sample analysis acquisition systems is presented in Ref. [17]. The suggested method used the average daily load history of the power transformer to estimate the insulator degradation; while the method's use of average data is advantageous, it deviates the health index estimation from its actual value in practice. Dissolved gas analysis (DGA) is a widely adopted diagnostic tool, with various techniques developed to improve fault detection. For instance, integration among DGA fault interpretation techniques such as Duval, Roger's four ratios, IEC methods, artificial neural networks (ANN), and conditional probability has demonstrated improved diagnostic accuracy compared to individual DGA methods [18,19]. These approaches not only address the limitations of individual diagnostic techniques but also enable early detection of faults, as evidenced by accuracy improvements which helps in determining a dependable transformer health index [20]. Solely traditional DGA-based diagnostics remain limited in specificity, often focusing on a single fault type or overlooking additional stress factors such as moisture, acidity, and breakdown voltage. For example, while techniques like the Duval Triangle and modified pentagons improve fault categorization, uncertainties persist when diagnosing multiple concurrent faults [19]. Methodologies that incorporate multi-parameter analyses, including health indices based on DGA and other oil properties, have shown promise in providing more comprehensive transformer evaluations.

Researchers [21,22] have explored artificial intelligence and machine learning techniques such as random forest support vector machine (SVM) and k Nearest Neighbor in addressing the missing data problem in assessing transformer condition and aging dependent failures. The authors in Ref. [23] established a method of predicting power transformer's insulation health index by applying missing data prediction techniques. It was noted that data availability is one way of ensuring the health index obtained is applicable even though, the assumption of missing data in this research was based on very few parameters, and also the computational complexity is intense and it worsen with increase parameters involved. The approach in Ref. [24] takes into account the impact of various crucial transformer parameters when assessing the general state of health, however it overlooks the key diagnostic tests associated with transformer insulation. Moreover, none of the models presented in Refs. [24,25] take into account how certain dissolved gases affect the transformers' health index.

While traditional methods like DGA provide valuable insights, a more comprehensive approach is necessary to assess the transformer's overall condition, especially the insulation system's health. Current transformer condition monitoring methods often focus primarily on DGA and oil diagnostics, but a more holistic approach that includes both oil and paper health assessments is required. This paper introduces the concept of Degree of Healthiness (DOH) and Degree of Faultiness (DOF), which are based on a wide range of attributes beyond just DGA. The new metrics provide a more comprehensive evaluation of transformer status, addressing the limitations of current diagnostic methods.

The paper proposes a score-based multi-parameter analysis technique DOH/DOF estimation model established upon an integrated fuzzy inference system by evaluating four key factors encompassing nineteen variables that influence the transformer condition and aging. The research utilizes the weighting factor based on Analytic Hierarchy Process (AHP) to evaluate the weights of the different variables under study to inform in rule formulation and prioritization of factors when mapping the DOH/DOF outcome within the fuzzy inference system. The overall outcome of the developed DOH/DOF model centers on entirely reflected attributes, not on any sole parameter. Furthermore, in this paper the transformer incipient faults are diagnosed based on the Duval Pentagon 1 (DP1) and Duval Pentagon 2 (DP2), combined Duval Pentagon 1 and Duval Pentagon 2 (CDP), and the modified combined Duval Pentagon (MCDP). The adopted Modified Combined Duval Pentagon fault diagnostic technique categorizes sections into regions of certainty and regions of uncertainty. This approach not only facilitates probable multiple fault identification, as proposed by Chatterjee et al. [26], but also addresses the rigidity associated with the pentagons and resolves discrepancies in fault detection when data from different sources are used [27].

### 1.2 Contributions and paper organization

i. Development of a DOH/DOF model for oil immersed power transformers based on fuzzy inference cumulative weighting grouped factor to ascertain transformer status. The assessment of the transformer status has taken into account the results of diagnostic tests for dissolved gases, the quality of the transformer oil, the integrity of the paper insulation, and the maintenance history.
ii. Establishment of a non-intrusive fuzzy inference model for the degree of polymerization estimation based on 2-FAL, carbon oxides ratio and ethanol that enables paper insulation interpretation.
iii. Integration of knowledge of incipient faults diagnosed based on Modified Combined Duval Pentagon alongside the DOH/DOF in interpretation transformer status gives a holistic way of looking at the complete asset as a whole.
iv. By developing fuzzy sub-models for each diagnostic factor, each sub-model provides a specific evaluation based on the respective diagnostic inputs, aiding in more precise maintenance planning.

The remainder of the paper is organized as follows: Section 2 introduces the DOH/DOF indicators. Section 3 explains the implementation of the DOH/DOF model, including subsections on the analogy behind the DOH/DOF, fuzzy inference model formulation, parameter score computation for the fuzzy degree of polymerization estimation model, DOH/DOF factor grouping, and incipient fault diagnosis. Section 4 presents the results and discussion, while Section 5 concludes the paper.

## 2 Degree of faultiness/degree of healthiness indicators

The critical identification and examination of the contributing factors that cause transformer deterioration is a prerequisite for developing a likely condition status (DOH/DOF) model for a power transformer. This section summarizes the key characteristics that impact the life span and insulation system degradation of power transformers in service, thus mappers of the DOH/DOF model.

### 2.1 Dissolved gas analysis (DGA)

Through different interpretation of DGA, seven extractable gases dissolved in oil can be used to ascertain the internal condition status of an oil immersed transformer. These gases include two carbon oxides; carbon monoxide (CO) and carbon dioxide (CO_2_); hydrogen (H_2_), and four hydrocarbon gases; ethylene (C_2_H_4_), methane (CH_4_), acetylene (C_2_H_2_), and ethane (C_2_H_6_) [5,13,21]. Notably, paper degradation-related faults are revealed by the manifestation of the carbon oxides, while C_2_H_4_ and C_2_H_6_ are important markers of oil thermal activity inside the transformer. The evolution of high quantities of C_2_H_2_ and H_2_ mirrors thermal arcing, whilst the accelerated increase in H_2_ and CH_4_ indicates partial discharge faults. This study quantified the concentration of dissolved gases using DGA data obtained through a Total Oil Gas Analyzer (TOGA) gas chromatography technique.

### 2.2 Oil quality (OQ)

Oil samples are subjected to a range of electrical, physical, and chemical tests in order to verify the quality of the oil. There are usually six commonly adopted attributes for use to ascertain the oil quality standing of an in-service power transformer. The techniques and benchmarks employed in this paper to quantify and ascertain the properties of the oil are underlined in Table 1 [28] while the parameter limits ranges are based on IEEE C57.106–2006 as highlighted in Ref. [24].

**Table 1:** Oil quality parameters.

Serial No	Oil Quality Parameter	Testing Method/Instrument	Standards
1	Break down voltage (kV)	Megger OTS100AF and Foster OSTS100F (0–100 kV)	IEC 60156
2	Dielectric Dissipation factor (DDF)	Oil Tan delta & Resistivity Test Kit Ex. MOTR	IEC 60247
3	Interfacial Tension (IFT)	Tensiometer	ASTM D-971
4	Water content moisture(ppm)	Karl Fischer Titration (KFT) method moisture-in-oil sensor	ASTM D1533
5	Acidity	Chemistry-neutralization method	ASTM D947
6	Colour and Appearance	Transmitted light and Visual inspection	IEC-ISO 2049 ASTMD-1524

### 2.3 Degree of polymerization (DP)

The degree of polymerization serves as a useful gauge of the transformer insulation paper's mechanical characteristics and state of deterioration [[24], [25], [26], [27], [28], [29]]. However, online measurements are not feasible for DP measurement since it is an invasive procedure that involves sample insulating paper from a disassembled transformer. Additionally, this method is costly, time-consuming, labor-intensive, and demands the highest attention. Due to this, new methods of DP estimate have been developed that use chemical indicators including furans, the CO_2_/CO concentration ratio, and the amount of methanol or ethanol in the oil [[30], [31], [32], [33]]. Moreover, computational models have been developed based on the correlation between 2-FAL concentration and DP values; a summary of these models can be found in Ref. [29]. Despite exclusive furan analysis being the most often used technique for estimating DP values in transformer insulation, its outcomes vary based on the insulation paper type, the loading condition, and the transformer's location. This study proposes an alternative approach of DP estimation, which has been discussed in the preceding sections.

### 2.4 Maintenance data

To evaluate the inclusive condition of a power transformer, it is essential to embrace as much data as is accessible and suitable for a realistic assessment. Maintenance data comes in different forms depending on the maintenance strategies utilities put in place in assessing their equipment and assets. In this paper, a ranking system was developed based on the maintenance work orders not done on routine basis but rather considered as diagnostic tests. The attributes considered are core to ground test, leakage reactance, turns ratio and winding resistance. The threshold for scoring limits for these variables are as in Ref. [24] based on IEEE Std 62™-1995. However, it is prudent to note that the test data for these variables may not be always available.

## 3 Transformer DOH/DOF estimation model

To begin with, the analogy of the degree of healthiness and faultiness of a power transformer is highlighted. The fuzzy inference tool was used to establish the transformer DOH/DOF estimation model based on the computation of unified cumulative attributes that signifies the transformer operational or deterioration status. These attributes are divided into four categories (factors), including degree of polymerization factor (DPF), dissolved gas (DGAF), oil quality (OQF), and maintenance data factor (MF). The fuzzy model inputs are based on the aggregation of each of the subsystem factors with respect to the totalized scores, weights, or impact on the transformer's insulation system state. The formulation of the fuzzy rule inference was based on the weighting of the categorized factors. The weight of each factor was calculated utilizing the analytical hierarchy process (AHP) method. The final aggregated DOH/DOF value is the linguistic output of the fuzzy logic model.

### 3.1 The degree of healthiness and Faultiness Analogy

**Fig. 1:**
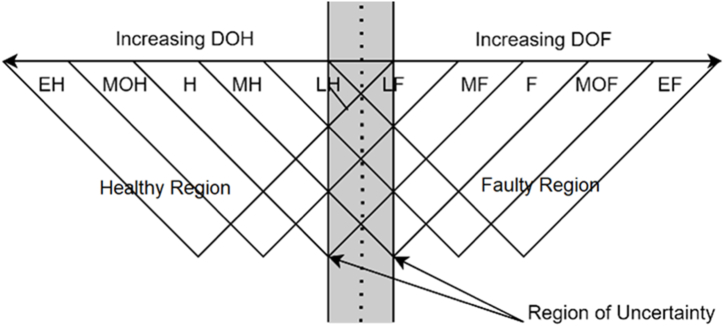
Degree of healthiness/degree of faultiness analogy.

The analogy is represented by a graphic depiction of a transformer's status trajectory at any particular instance, as illustrated in Fig. 1. The transformer's condition is divided into categories, healthy and faulty regions, each with discrete bands labeled with a linguistic marker. Transformers at peak health are represented by the Extremely Healthy (EH); however, the health condition reduces in the successive bands. At the center there exists an overlap area, a region of uncertainty marking the transition from healthy to faulty transformer due to degradation or ageing. This may be so since the degree of healthiness and faultiness are not rigid values but rather ongoing and continuous phenomena affected by the errors in the measuring of attributes used to compute them. In the faulty region, the transformer's condition successively deteriorates towards the right, with the ultimate being Extreme Faulty (EF), indicating a high level of deterioration, with the transformer at significant risk of failure. This DOH/DOF analogy is also realized in Ref. [4].

### 3.2 Fuzzy inference DOH/DOF model formulation

The transformer degree of healthiness/faultiness model integrates the data obtained from numerous attributes of transformers in service. The established DOH/DOF model inputs are founded on the concentration of dissolved gases factor score (DGAF), oil quality factor score (OQF), degree of polymerization factor score (DP), and the maintenance data factor score (MF). DGAF encompasses seven variables, OQF involves six attributes, DP has three attributes while MF consists of four parameters. In the present model, the DP is a result of the estimated value from an established alternative fuzzy inference model in which furan content, carbon oxide ratios and ethanol were the inputs. The parameter ranges that power utilities can reasonably adopt have been defined by the IEEE, IEC, Doernenburg, California State University Sacramento, and Bureau of Reclamation [34]. In the same notion, the standards adhered to can affect the transformer's DOH/DOF prediction. To realize a practical DOH/DOF value from a fuzzy logic inference system, normalization of inputs was accomplished by using parameter limits that signifies normal and extreme ranges adopted from Ref. [28]. Accordingly, the four clusters of each variable (factor) are allotted appropriate weights (*w*) on a scale spanning between 0 and 10 as tabulated in Table 2.

•**Parameter Score Computation**

**Table 2:** Weight classification for the four variable classes.

Description	Condition	Weight (*w*)
Claster A	Good	[0 ≤ w ≤ 2.5]
Claster B	Acceptable	[2.5 < w ≤ 5.0]
Claster C	Poor	[5.0 < w ≤ 7.5]
Claster D	Worst	[7.5 < w ≤ 10]

Computation of the exact score of each individual parameter was based on the quantified value from the experiments and observation data of different in-service transformers and keyed in expression shown in equations [1], [2] adopted from Refs. [28,34].

[1] $$\text{Variable}\;\text{score}=k_{i}+\left[\left(\frac{x_{i}-y_{i}}{z_{i}-y_{i}}\right)\times 2.5\right]$$

[2] $$\text{Variable}\;\text{score}=k_{i}+\left[\left(\frac{z_{i}-x_{i}}{z_{i}-y_{i}}\right)\times 2.5\right]$$

*i* = 1,2, 3 … n, where *n* is the number of variables being considered in the factor. $k_{i}$ = minimum weight in the four settings of the variables. $x_{i}$ is the current measured value. $z_{i}$ and $y_{i}$ are the upper and lower limits of the matching group of the variables. $\frac{x_{i}-y_{i}}{z_{i}-y_{i}}$ represents the assigned inputs' normalized expression governed by Ref. [1] whose lower values are desired, whereas variables that symbolizes heathy conditions by having higher values are normalized by $\frac{z_{i}-x_{i}}{z_{i}-y_{i}}$ and the score evaluated by expression of assigned inputs governed by Ref. [2].

### 3.3 DOH/DOF factor grouping (sub model formulation)

The extend to which the power transformer is healthy or faulty is evaluated holistically based on the following identified main factors that influence the operation and ageing of a power transformer. The identified attributes are clustered into factors that by aid of fuzzy inference a cluster score is mapped into outcome which either fall in the healthy or faulty region of a transformer status. Thus, the following section outlined the roadmap in formulation of the sub models of the factors leading to an integrated DOH/DOF transformer model.

i. Degree of Polymerization Estimation Model

**Fig. 2:**
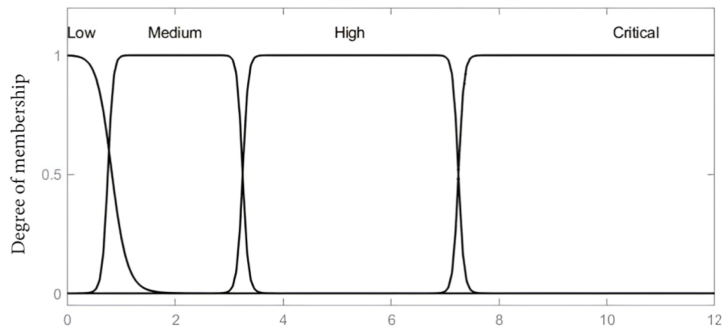
Input parameter membership function-furans (2-FAL) (ppm).

**Fig. 3:**
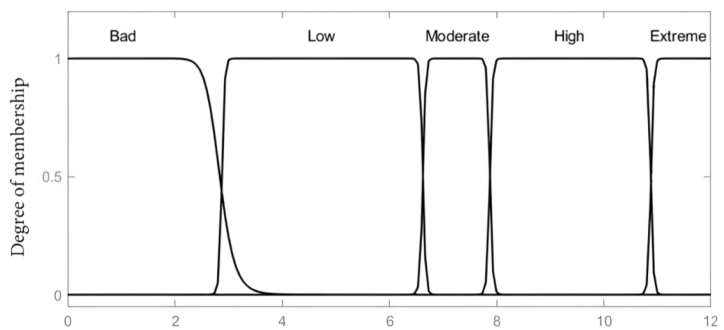
Input parameter membership function-CO2/CO ratio.

**Fig. 4:**
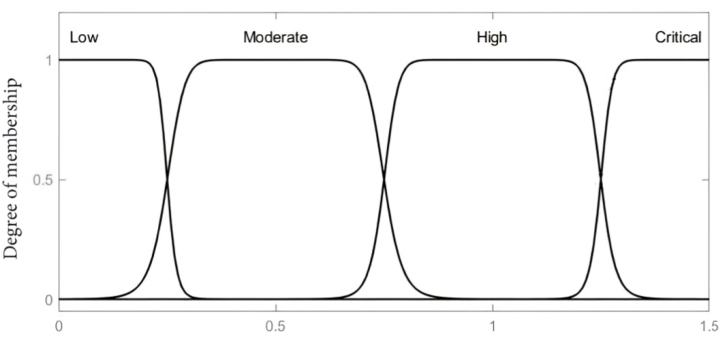
Input parameter membership function-MeOH (ppm).

**Fig. 5:**
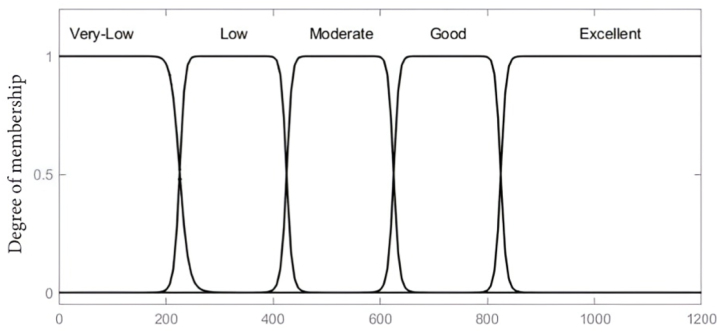
Output variable membership function-DP value.

The main byproducts of transformer solid insulation degradation considered in this paper to mirror the DP value of paper insulation constitute of furans (2-FAL), CO_2_, CO and methanol (MeOH) concentration levels. These variables; 2-FAL, MeOH and CO_2_/CO serve as the inputs to the fuzzy inference model, with the output being the estimated insulation DP value. The associated membership functions as input variables are set based on the concentration of furan (2-FAL) considered on a scale of 0–12 (ppm), CO_2_ and CO as a ratio from 0 to 12 and MeOH on a scale of 0–1.5 (ppm). The gauss bell membership linguistic labels considered for the furan input variable are low, medium, high and critical, whilst CO_2_/CO input variable uses bad, low, moderate, high and extreme whereas low, moderate, high and critical were adopted for MeOH as depicted in Fig. 2, Fig. 3, Fig. 4. The Gaussian membership function was selected due to its smoothness, parameter efficiency, robustness to noise, and its established use in fuzzy logic systems for nonlinear applications. These characteristics align with the requirements of the developed model for representing model variables effectively. The centroidal defuzzification technique mapped the membership functions of the outcome signifying the DP value on a scale of 0–1200, where linguistic label of Very Low, Low, Moderate, Good, and Excellent are used as indicated in Fig. 5.

Fuzzy rules in form of *“IF-THEN”* statements relating the input variables to the output were established based on transformer test data interpretation, some of the formulated rules include.

1. If (2_FAL-(ppm) is Low) and (Carbon-Oxide-Ratio is Low) and (MeOH_(ppm) is Low) then (DP-Value is Excellent)
2. If (2_FAL-(ppm) is Low) and (Carbon-Oxide-Ratio is Bad) and (MeOH_(ppm) is Moderate) then (DP-Value is Moderate)
3. If (2_FAL-(ppm) is Moderate) and (Carbon-Oxide-Ratio is Moderate) and (MeOH_(ppm) is Low) then (DP-Value is Good)
4. If (2_FAL-(ppm) is Low) and (Carbon-Oxide-Ratio is Bad) and (MeOH_(ppm) is High) then (DP-Value is Low)

**Fig. 6:**
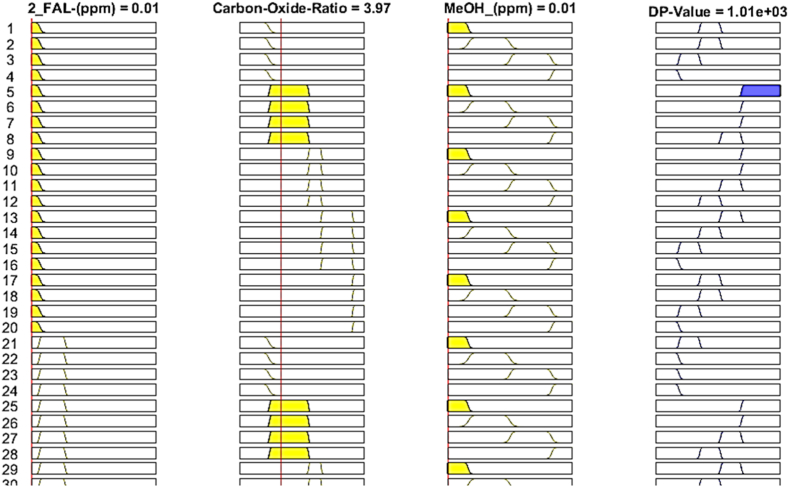
Rules-Viewer for the established DP model.

**Fig. 7:**
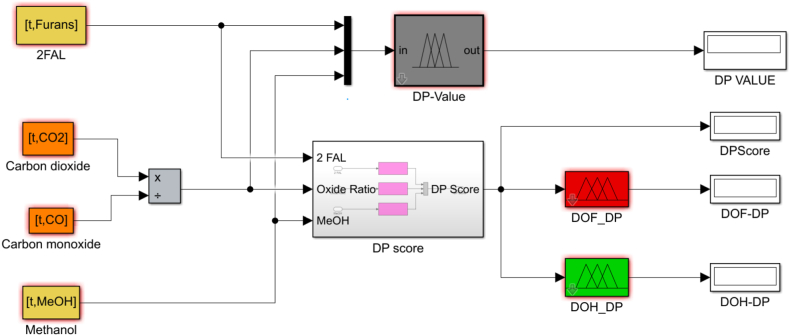
The Simulink model for the Degree of polymerization estimation & *DOH/DOF DPF model.*

In Fig. 6, the established rules associated with the developed DP estimation model are graphically represented. The Simulink sub-model for the developed fuzzy inference system illuminating the link between input and outcome for the DP value is portrayed in Fig. 7. For illustration purposes, the model is tested to establish the insulation DP value with inputs such as Furans (0.01 ppm) and CO_2_ (1020 ppm) and CO (257 ppm) and MeOH (0.01 ppm) as detected in one of the in-service power transformers. The model result indicates an excellent status of the solid insulation (paper) with a DP value of 1015, as shown in Fig. 6, Fig. 7.

•***Degree of Polymerization Factor (DPF)***

**Fig. 8:**
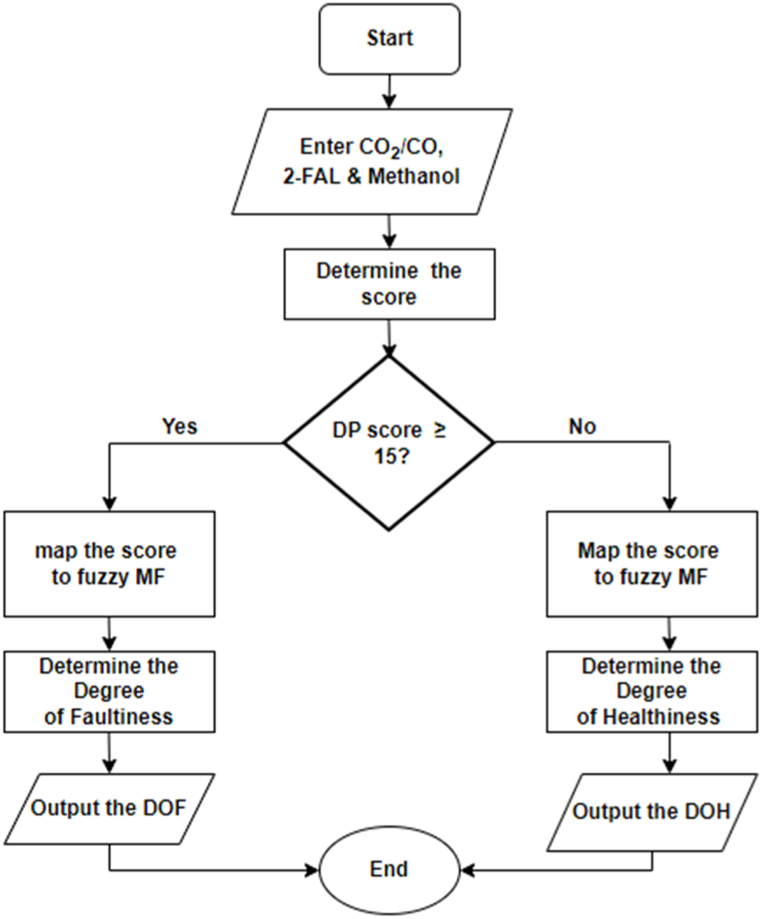
Degree of polymerization factor flow chart.

The DP factor enables aid in determining the transformer DOH/DOF because it represents three crucial properties of the cellulose paper in power transformers: mechanical strength, electrical insulation, and thermal properties. Three inputs—2-FAL, CO2/CO, and MeOH—as established in the developed fuzzy DP sub-model are used in the calculation of a score that will be mapped in establishing the region in which the solid insulation falls. Since the inference is based on fuzzy logic, the computation of the range for the membership was achieved by multiplying the lower and upper limits of weights of the four clusters (Table 2) by the number of variables in each grouping; in this case, the span of the score ranges from 0 to 30. A midpoint score of 15 serves as a turning point from region of healthiness to region of faultiness. The procedure followed in establishing the DPF fuzzy sub-model is highlighted in Fig. 8, while Fig. 7 also shows the combined formulated fuzzy DP estimation model and the DOH/DOF DPF sub-model.

ii. Dissolved Gas Analysis Factor and Incipient Fault Diagnosis

**Fig. 9:**
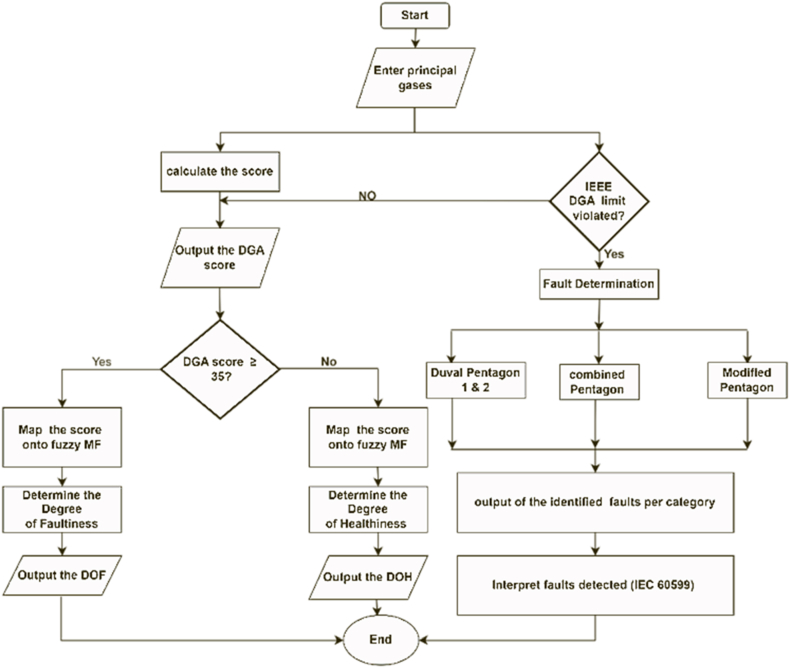
DGAF and fault diagnosis flow chart.

**Fig. 10:**
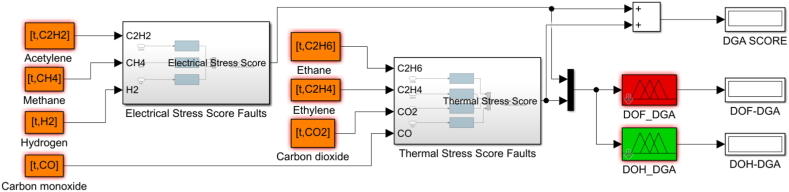
DOH/DOF sub model based on DGAF.

The DGAF is evaluated by considering the combustible gases dissolved in transformer oil. H_2_, C_2_H_2_, CH_4_, being indicators of electrical faults in oil are grouped together to give the electrical stress score while C_2_H_4,_ C_2_H_6_, CO_2_ and CO are grouped together to give the thermal fault score. An equal priority in fuzzy rules is given to both the electrical and the thermal stress scores. Because seven variables were involved in determining the total score, the span of the score ranges from 0 to 75. A midpoint score of 35 serves as a turning point from region of healthiness to region of faultiness. The incipient fault diagnosis was based on five hydrocarbon gases evolved in the transformer oil in determination of the various incipient faults that might have occurred. The gas concentrations are checked whether they are violated beyond the minimum recommended values by IEC C57.102. Once the minimum gas concentration requirements have been violated, that would imply there's a fault or accelerated degradation in the transformer's insulation system. This research therefore utilizes the Duval Pentagon 1 & 2, Combined Duval pentagon (CDP) and modified Duval Pentagon (MCDP) methods in diagnosing the incipient faults. The use of different methods increases the accuracy of fault detection and also gives more highlights on the most probable multi-faults that might be occurring in the power transformer by eliminating the rigidity associated with the exact boundaries in case of overlapping detected faults. Fig. 9 shows the steps involved in obtaining the DGAF and the fault diagnosis pathway whilst Fig. 10 depicts the Simulink sub model of the DOH/DOF based on DGAF.

•**Incipient Fault Diagnosis.**

**Fig. 11:**
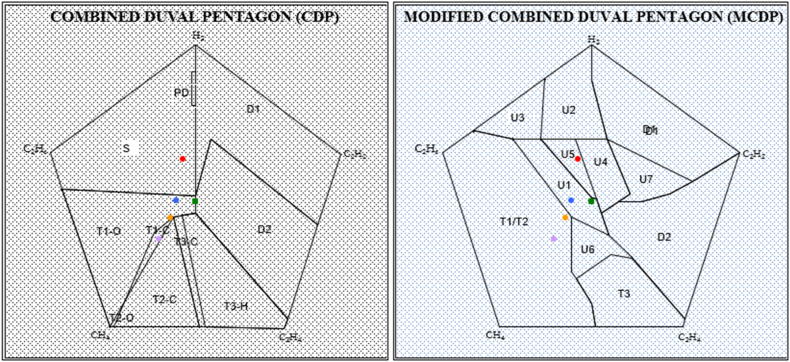
The established CDP and MCDP for fault diagnosis.

The fault detection based on Duval pentagon was first developed by Michel Duval [35]. The method utilizes relative percentage concentration of five hydrocarbon gases; H_2_, C_2_H_2_, CH_4_, C_2_H_4,_ C_2_H_6_ in graphical fault identification. For each gas, zero (0 %) of the concentration is plotted at the center while one hundred (100 %) is plotted at the apex [36]. The five hydrocarbon gases are entered and through the equations shown in Refs. [14,34,37], the centroid of the combinations is plotted in the pentagon. The established combined Duval Pentagon (CDP) coordinates are as in Table 3 showing an overlapping of Duval Pentagon 1 and Duval pentagon 2. This creates three more regions as opposed to the seven regions of the individual Pentagons; T2-O, T1-C and T3-C. This facilitates easy possible fault combination detection and simplifies the need of having two different pentagons. Similarly, the adopted modified Combined Duval pentagon (MCDP) uses the same principle but with different coordinates as shown in Table 5 [23]. The zone in which the centroid lies indicate the incipient fault in the transformer and interpreted based on IEC 60599 as in Table 4, Table 6 [14]. The established combined Duval pentagon (CDP) and modified combined Duval pentagon (MCDP) based on coordinates show cased in Table 3, Table 4 are depicted in Fig. 11.

iii. Oil Quality Factor

**Table 3:** Combined Duval Pentagon (CDP) coordinates.

Zone	Coordinates
S	(0, 40), (−38, 12.4), (−35, 3), (0, 1.5), (0, 24.5), (−1, 24.5), (−1, 33), (0, 33), (0, 40)
T1-O	(-21.5, −32), (−11, −8), (−6, −4), (0, −3), (0, 1.5), (−35, 3), (−22.5, −32), (−21.5, −32)
T2-C	(1, −32), (−6, −4), (−22.5, −32), (1, −32)
T3-H	(24, −30), (0, −3), (−3.5, −3.5), (2.5, −32), (23.2, −32.4), (24, −30)
D2	(4, 16), (32, −6), (24, −30), (0, −3), (0, 1.5), (4, 16)
D1	(0, 40), (38, 12), (32, −6), (4, 16), (0, 1.5)
PD	(0, 24.5), (−1, 24.5), (−1, 33), (0, 33), (0, 24.5)

**Table 4:** CDP Incipient Fault Key identification.

Zone	Incipient Fault
S	Stray Gassing
T1-O	Thermal fault <300 °C in oil
T2-C	Thermal Fault 300 °C–700 °C with possible paper carbonization
T3-H	Thermal Fault of >700 °C in oil only
D2	High Energy Discharge
D1	Low Energy Discharge
PD	Partial Discharge
T1-C	Thermal fault at temperature <300 °C with possible paper carbonization
T2-O	Thermal Fault 300 °C–700 °C with less likelihood of paper involvement
T3-C	Thermal Fault affecting paper insulation oof temperature >700 °C

**Table 5:** MCDP with Region of Certainty and Region of uncertainty.

Zone	Coordinates
T1//T2	(-30.29, 18), (−20, 16), (−5, −4), (−5, 18), (−3.9, −19.76), (0, −26), (1, −32), (−23.5, - 32), (−38, 12.4) (−30.29, 18)
T3	(-3.9, −19.76), (0, −26), (1, −32), (23.5, −32), (24, −30), (10.33, −14.5), (5, −13.7), (−3.9, −19.76)
D2	(38, 12.4), (26, 5), (20, 2), (13, 0), (7, 0), (2.5, −3), (4.5, −8.75), (10.33, −14.45), (24, −30), (38, 12.4)
D1	(38, 12.4), (26, 5), (4, 16), (0, 31), (0, 40), (38, 12.4)
U1	(-20, 16), (−13,16), (0, 1), (1.3, 0.56), (2.5, −3), (4.5, −8.75), (−5, −4)
U2	(-13, 16), (4, 16), (0, 31), (0, 40), (−12, 31.28)
U3	(-30.29, 18), (−20, 16), (−13, 16), (−12, 31.28), (−30.29,18)
U4	(-4, 16), (4, 16), (10, 2), (7, 0), (2.5, −3), (1.3,0.56)
U5	(-13, 16), (−4, 16), (1.3, 0.56), (0, 1), (−13, 16)
U6	(-5, −4), (−5, −18), (−3.9, −19.76), (5, 13.7), (10.33, −14.45), (4.5, −8.75), (−5, −4)
U7	(4, 16), (10, 2), (7, 0), (13, 0), (20, 2), (26, 5)

**Table 6:** MCDP Incipient fault zones.

Region	Certain Fault	Region	Probable fault
PD	Partial Discharge	U1	T1/T2, PD, D2
D1	Low Energy Discharge	U2	PD, D1
D2	High Energy Discharge	U3	T1/T2, PD
T3	Thermal Fault >700 °C	U4	PD, D1, D2
T1/T2	Thermal Fault <700 °C	U5	PD, D1, D2, T1/T2
		U6	D2, T1/T2, T3
		U7	D1, D2

**Fig. 12:**
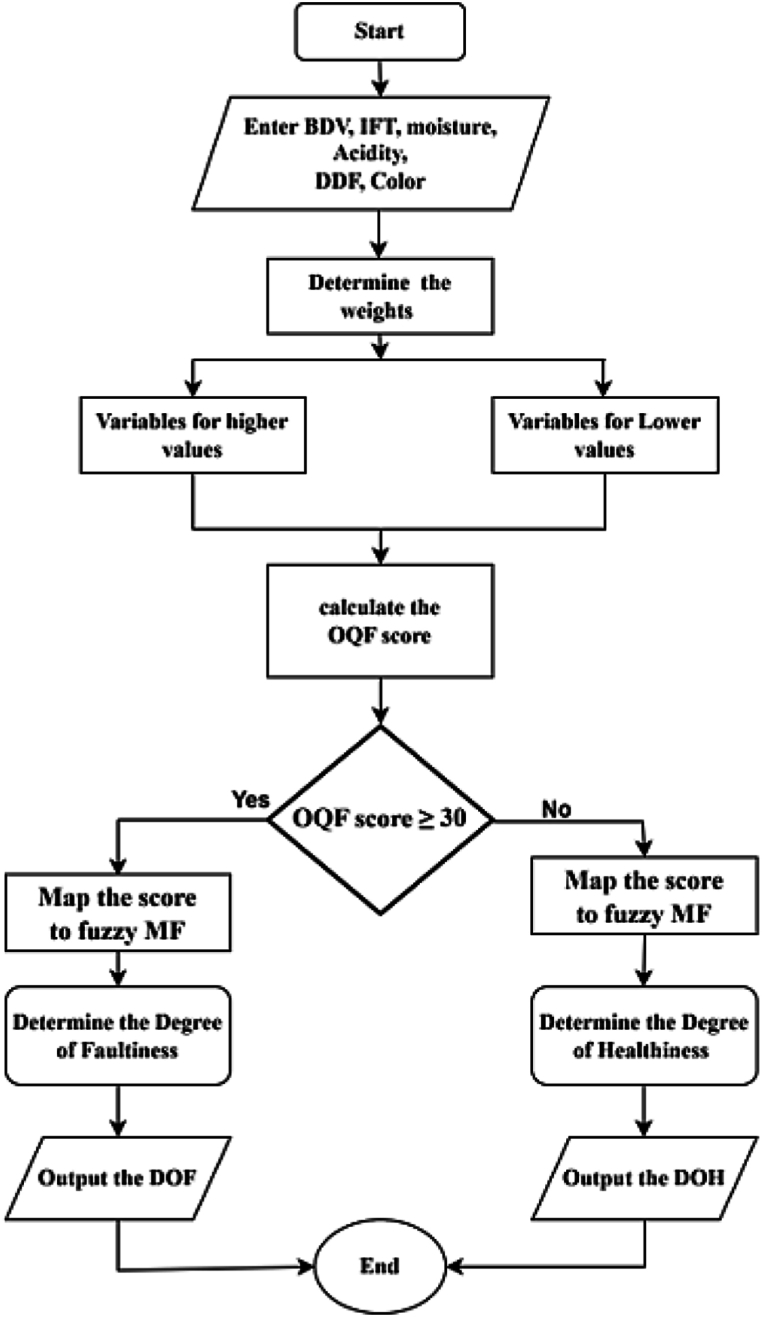
The Oil Quality Factor flow chart.

**Fig. 13:**
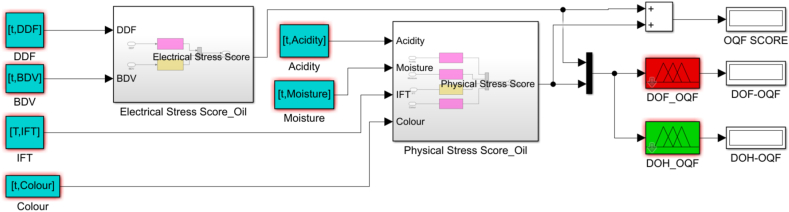
DOH/DOF based on OQF.

The Oil Quality Factor (OQF) is an indicator of the effectiveness of the transformer oil to perform its two main functions; electrical insulation-preventing contacts between components of different electrical potentials and cooling-absorbing heat and propagating it to external cooling systems. The inputs are categorized in two electrical stress score comprising of breakdown voltage and dielectric dissipation factor representing the electrical properties of oil and physical stress score comprising of acidity, moisture, interfacial tension and colour representing the physical properties of transformer oil. On a universe of discourse of 0–60 a midpoint of 30 marks transition from region of healthiness to faultiness of the transformer oil insulation system. Four membership functions with labels medium faulty (MF), faulty (F), more faulty (MOF) and extremely faulty (EF) describe the input score evaluating the DOF whilst medium healthy (MH), healthy (H), more healthy (MOH) and extremely healthy (EH) labels describe the membership functions that map the DOH. Procedure for achieving the DOH/DOF based on OQF is shown in Fig. 12 and the established fuzzy sub model is depicted in Fig. 13.

iv. Maintenance Factor

**Fig. 14:**
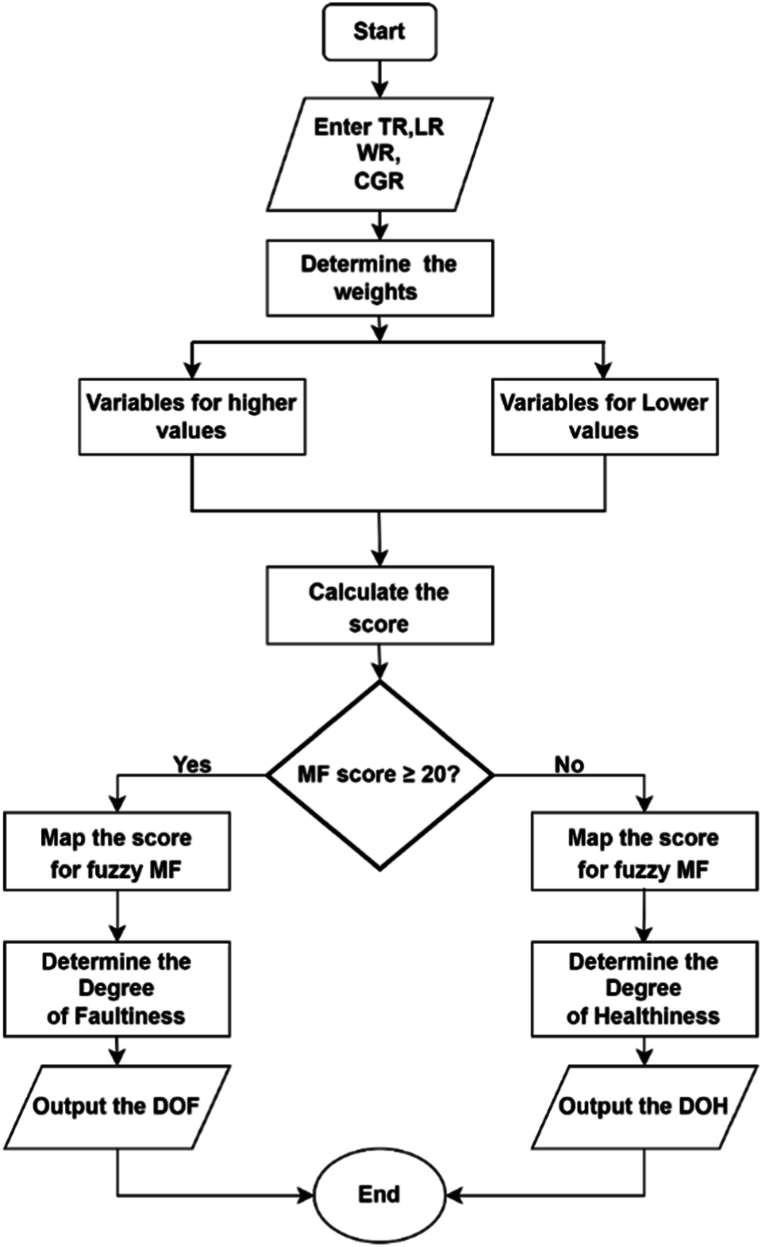
The Maintenance Factor flow chart.

**Fig. 15:**
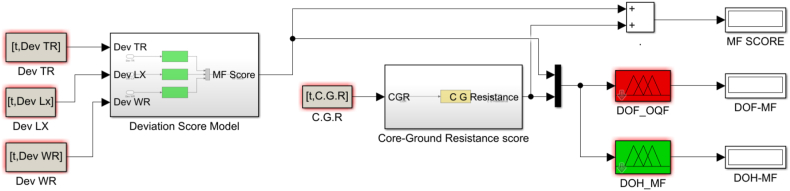
DOH/DOF based on MF.

The Maintenance Factor (MF) is a representation of effort and resources needed to maintain a transformer operating at optimum conditions. MF changes with time as the transformer ages as well as the operating conditions. In Fig. 14, the inputs are categorized in to deviation score comprising of leakage reactance, turns ratio and winding resistance while the core to ground resistance being a standalone. On a universe of discourse of 0–40 a midpoint of 20 marks the evaluation of DOH or DOF of the particular transformer. Four membership functions with labels medium faulty (MF), faulty (F), more faulty (MOF) and extremely faulty (EF) describe the input score evaluating the DOF whilst medium healthy (MF), healthy (H), more healthy (MOH) and extremely healthy (EH) labels describe the membership functions that map the DOH. In all the cases both the DOH and DOF are mapped on a scale of 0–1 which can be interpreted in percentage form. Procedure for establishing the sub model for DOH/DOF based on maintenance factor is shown in Fig. 14 and the established fuzzy sub model is highlighted in Fig. 15.

### 3.4 Overall DOH/DOF fuzzy inference model

•**Parameter Weighting**

Transformers in-service can suffer from electrical, chemical, and/or thermal stress that can accelerate their degradation, leading to premature failure before reaching the technical design life span. The overall DOH/DOH transformer status was arrived at after integrating the fuzzy sub-models of the grouped factors. Since fuzzy inference was used in establishing the overall model based on the different group variables scores of DGAF, DPF, OQF, and MF, rule formulation was founded on weights calculated so as to establish which factor to prioritize. The Analytic Hierarchy Process (AHP) method was adopted in the computation of weights due to its ease of use and effectiveness in multi-criteria decision-making. The eigenvector method was employed, where each row of the normalized matrix was multiplied by its corresponding column sum. The elements in each row were then averaged to obtain the initial weights, which were subsequently normalized to yield the final weights. This study utilizes a balanced AHP scale rather than a linear one to account for the uneven distribution of local weights, which can reduce sensitivity when assessing closely related elements [38,39]. From the computation outcome, DGA had a higher weight based on its criticality to transformer health condition and has been widely used as one of the measurements for evaluating the condition of the power transformer oil. It is closely followed by DP, an indicator of cellulose insulation paper condition. The oil quality factor and the maintenance factor had the least weights, as noted in Table 7. A 10 % threshold for the consistency ratio (CR) was adopted, indicating that the results are reliable in terms of consistency, as recommended in the literature [40,41].

•**Cascaded DOH/DOF Fuzzy Inference Model**

**Table 7:** Factor weights.

Variables	Factor	Calculated Factor weight
Furans; CO, CO_2_, MeOH	DPF	29.07
Acidity; Moisture; Interfacial Tension; Colour; BDV; DDF	OQF	20.27
H_2_; CH_4_; C_2_H_4_; C_2_H_6_; C_2_H_2_; CO_2_; CO	DGAF	35.83
Turns ratio; Leakage reactance; Winding Resistance; Core to Ground Resistance	MF	14.83

**Fig. 16:**
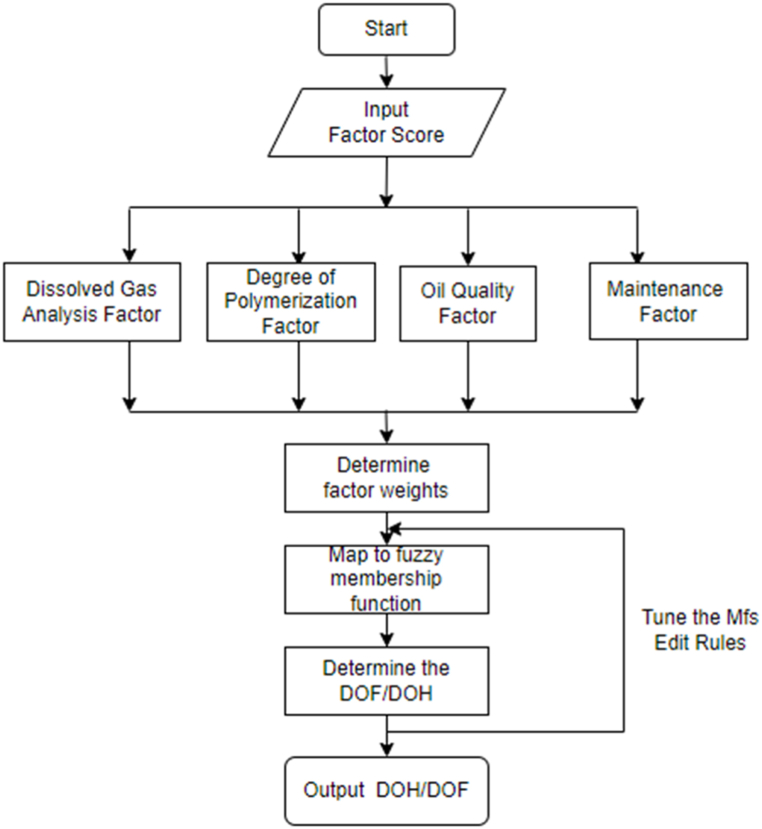
Overall DOH/DOF flowchart.

To achieve the overall transformer DOH/DOF model, steps in Fig. 16 are followed. The final inputs to the DOH/DOF model were the outcome of sub-models of the cascaded models of (DGAF, OQF and DPF) and MF in which their membership functions were partitioned on a scale of 0–1. Thus, a final two-input (incipient factor and MF) one-output model mapped the DOH/DOF outcome. The incipient factor is the output of the integrated sub-models of DGAF, OQF and DPF. The output of the overall model has Medium Faulty (MF), Faulty (F), More Faulty (MOF) and Extremely Faulty (EF) as the labels for the gauss bell membership functions that map the DOF, whilst Medium Healthy (MH), Healthy (H), More Healthy (MOH) and Extremely Healthy (EH) labels describe the gauss bell membership functions that map the DOH. Some of the fuzzy *“IF-THEN”* rules mapping overall DOH/DOF include:

1. If (INCIPIENT-DOH is LH) and (MF-DOH is LH) then (Tx-DOH is LH)
2. If (INCIPIENT-DOH is LH) and (MF-DOH is MH) then (Tx-DOH is MH)
3. If (INCIPIENT-DOF is LF) and (MF-DOF is LF) then (Tx-DOF is LF)
4. If (INCIPIENT-DOF is LF) and (MF-DOF is MF) then (Tx-DOF is MF)

**Fig. 17:**
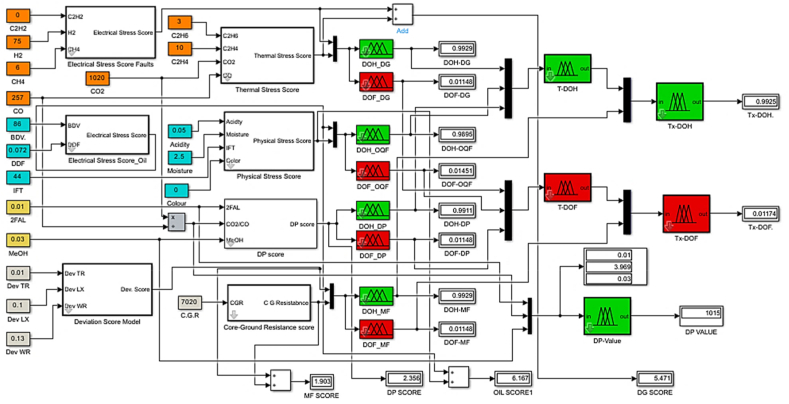
Overall transformer DOH/DOF Simulink model.

**Fig. 18:**
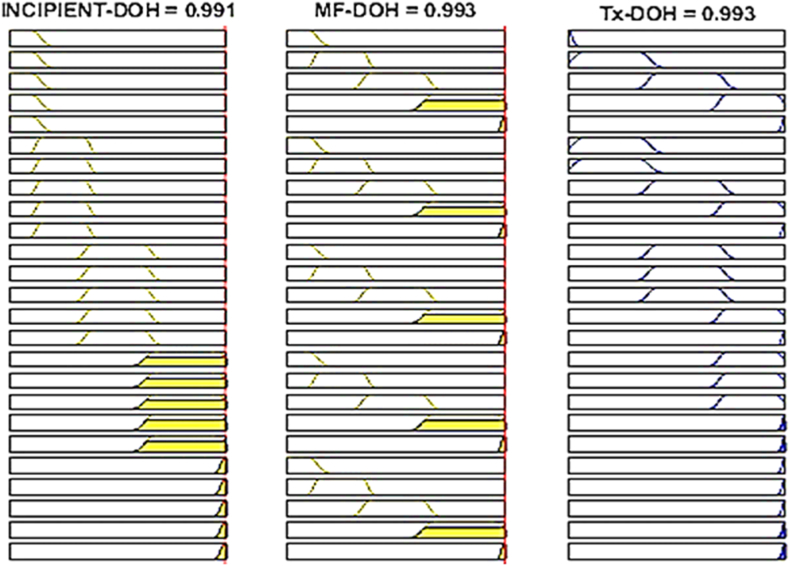
Rules-Viewer for the established DOH model.

**Fig. 19:**
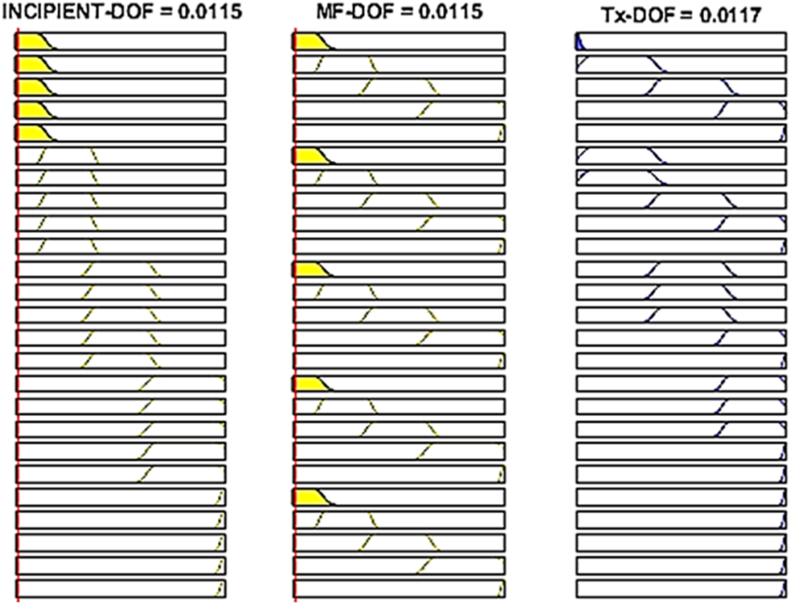
Rules-Viewer for the established DOF model.

The proposed fuzzy logic transformer DOH/DOF estimation that can map the transformer status is depicted in Fig. 17, whilst the overall transformer DOH and DOF for different sets of inputs can also be interpreted from a fuzzy rule surface viewer as shown in Fig. 18, Fig. 19 respectively.

## 4 Results and discussion

To evaluate the validity and accuracy of the proposed fuzzy logic inference methodology for estimating the DP, the incipient faults, and the overall DOH/DOF, power transformer data obtained from power utility companies in South Africa and Zimbabwe were used as test input variables. The data-set includes information from 95 transformers with various service life spans, capacities, loading patterns, and cooling mechanisms (ODAF- Oil Directed Air Forced, ONAN- Oil Natural Air Natural, ONAF- Oil Natural Air Forced). For validation purposes, Table 8a, Table 8b(a) and 8(b) presents sample data from 15 representative transformers, selected to reflect the diversity of the larger data-set. The transformers in Table 8(a) are the same ones in Table 8(b), only the difference in the data set obtained. In totality around twenty-two different attributes were used in validation on the model. The observed results for the developed models giving an outcome of fault diagnosis, DP estimation and finally the DOH/DOF are presented in the subsequent subsections.

i. Incipient Fault Diagnosis Results

**Table 8a:** Test data I.

*Tx No*	*Year of Installation*	*H*_*2*_*ppm*	*CH*_*4*_*ppm*	*C*_*2*_*H*_*2*_*ppm*	*C*_*2*_*H*_*6*_*ppm*	*C*_*2*_*H*_*4*_*ppm*	*CO*_*2*_*ppm*	*CO ppm*	*O*_*2*_*ppm*	*Furans ppm*	*Moisture ppm*	*MeOH ppm*
Tx1	1996	188	300	30	56	29	3611	300	8865	0.25	22	0.25
Tx2	2010	15	8	0	9	5	7135	902	1672	0.1	10	0.21
Tx3	2012	793	704	1	8	18	1873	245	5300	0.03	9	0.8
Tx4	1987	82	344	37	202	162	22589	8197	20971	4.53	28	2.2
Tx5	1998	151	8	8	151	10	2323	297	14620	0.22	6	1.02
Tx6	2000	107	129	0	55	68	7038	892	5013	0.1	26	0.25
Tx7	2015	102	38	3	8	15	1077	165	679	0.02	3	0.2
Tx8	1978	1498	395	26	323	395	12371	1582	25623	3.9	33	80
Tx9	2005	1176	4	1	4	10	4991	637	3892	0.83	10	0.81
Tx10	2002	181	79	21.1	56	29	713	196	10032	2.91	21.2	2.9
Tx11	2018	75	6	0	3	10	1020	257	598	0.01	2.5	0.01
Tx12	1996	185	79	20	120	195	3100	430	3900	1.2	212	0.8
Tx13	1990	1498	395	26	323	395	12260	1475	26630	2	24	0.83
Tx14	1984	1400	320	20	148	395	7038	900	21430	1.2	17	1
Tx15	1993	185	315	29	56	29	3622	471	19340	3.03	22	0.38

**Table 8b:** Test data II.

*Tx No*	*Year of Installation*	*Acidity mg KOH/g*	*BDV kV*	*DDF* *%*	*IFT dynes/cm*	*Colour*	*WT*^*o*^*C*	*Turns Ratio (TR) Δ%*	*Leakage Reactance (Δ%)*	*Core to Ground Resistance (GΩ)*	*Winding Resistance (Δ%)*	*DP*
Tx1	1996	0.07	52	0.14	29	1.5	60	0.35	0.48	6.56	0.58	650
Tx2	2010	0.03	55	0.15	42	1	61	0.01	0.67	5	0.56	750
Tx3	2012	0.04	71	0.068	45	0	56	0.023	0.21	5.82	0.22	840
Tx4	1987	0.18	40	0.266	20	2.5	78	1.33	1.78	0.74	2.44	220
Tx5	1998	0.13	64	0.566	27	2	68	0.52	0.33	4.6	1.08	680
Tx6	2000	0.09	48	0.249	25	1.5	66	0.04	0.56	2.3	0.84	780
Tx7	2015	0.01	82	0.083	45	0	60	0.02	0.36	5.02	0.3	1030
Tx8	1978	0.19	35	0.593	25	4	66	1.02	2.81	0.55	3.1	260
Tx9	2005	0.09	62	0.733	29	1.5	53	0.35	1.33	2.2	1.2	600
Tx10	2002	0.349	33.5	0.257	20	3	50	0.63	–	1.53	0.98	375
Tx11	2018	0.05	86	0.072	44	0	52	0.01	0.1	7.02	0.13	1090
Tx12	1996	0.08	50	0.257	20	3	54	0.08	2.5	0.025	1.13	470
Tx13	1990	0.18	33	0.593	20	2.1	60	1.2	2.80	0.009	3.1	360
Tx14	1984	0.06	35	0.593	25	1.7	66	1.01	2.71	0.198	3.1	350
Tx15	1993	0.07	52	0.14	25	2	61	0.8	2	0.85	1.5	500

The fault identification was done based on Duval pentagon methodology in which only the outcome of combine Duval Pentagon (CDP) and Modified Combined Duval Pentagon (MCDP) results are presented in this paper. Variety of fault cases of visually inspected and verified 65 faulted transformers were used for substantiation and comparison among the applied technique. Table 9, Table 10 provides an inclusive overview of the total diagnostic accuracy of the incipient faults. For the cases evaluated, it is evident that the Modified Duval Pentagon performs better than the Combined Duval Pentagon method using data in Ref. [42], while focusing on power transformers without communicating OLTC and Power transformers with communicating OLTC. Thermal faults of below 700 °C had the lowest accuracy as most of the point were detected as thermal faults of high temperature T3. Improved accuracy of the MCDP is attributed to its multiple fault detection capability and robustness.

**Table 9:** Fault detection based on the Combined Duval Pentagon (CDP).

Incipient fault type	Number of physically Inspected cases	Correctly detected	Wrongly detected	Wrongly detected as
PD	9	8	1	1 Stray gassing (S)
D1	10	9	1	D2
D2	26	23	3	2 D1 & 1 S
T1/T2	10	4	6	4 T3, 2 S
T3	10	9	1	1 T2
**Total**	**65**	**53**	**12**	**12**

**Table 10:** Fault detection based on the Modified Combined Duval Pentagon (MCDP).

Incipient fault type	Number of physically Inspected cases	Correctly detected	Wrongly detected	Wrongly detected as
PD	9	9	0	0
D1	10	10	0	0
D2	26	25	1	1 D1
T1/T2	10	9	1	1 T3
T3	10	9	1	1 T1/T2
**Total**	**65**	**62**	**3**	**3**

A comparison summary in terms of percentage of the employed technique efficiency of the total 65 cases considered is given in Table 11.

**Table 11:** Percentage comparison summary.

Technique	Efficacy (%)	Wrong detection (%)
Combined Duval Pentagon (CDP)	81.5	18.5
Modified Combined Duval Pentagon (MCDP)	95.4	4.6

**Fig. 20:**
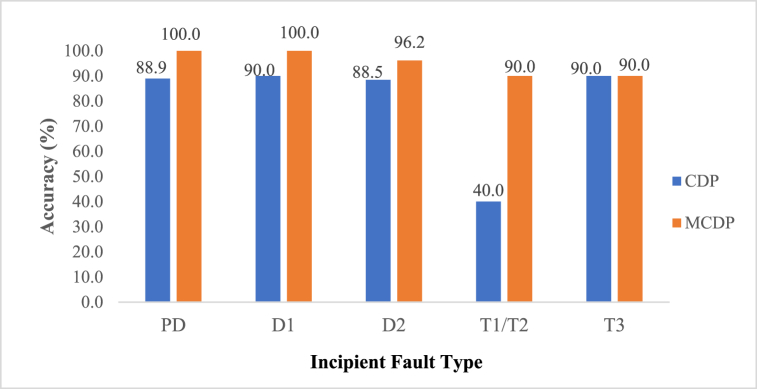
Incipient fault diagnosis accuracy.

The accuracy of each technique per incipient fault detected in percentage, is then evaluated by taking the overall number of faults cases correctly detected divided by the total number of cases under consideration multiplied by 100 %. The higher the efficacy, the better the technique in detecting the incipient fault and vice versa. The accuracy comparison of the two techniques is shown in Fig. 20 in form of comparative bar graphs per each fault.

ii. Degree of Polymerization (DP)

**Fig. 21:**
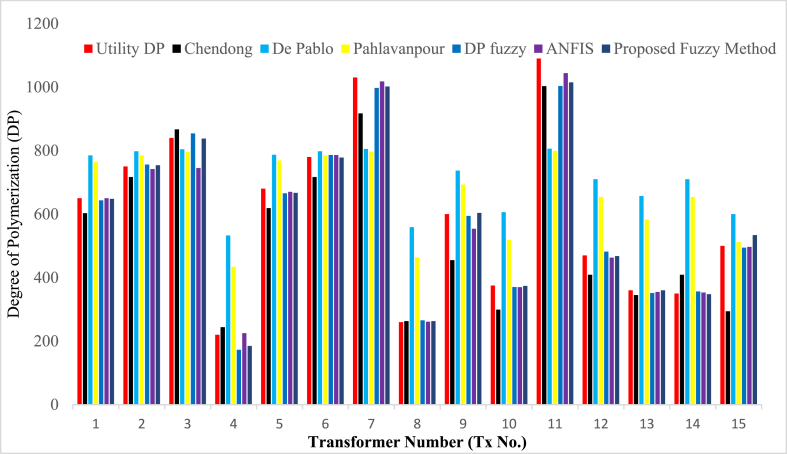
Comparisons of DP values from different models.

In this paper, DPs measured values from utility company for several transformers have been used to evaluate the performance of the developed fuzzy model. Results of the assessment of the state of cellulose paper by the proposed non-intrusive fuzzy logic model was comparable the with those of the insulation paper predicted by mathematical models and measured DP values by the utility. Table 12 shows the comparison of obtained DP results of the developed fuzzy logic inference model and those of mathematical models, ANFIS and fuzzy logic models based on the test data for 15 transformers presented in Table 8a, Table 8b(a) and 8(b). Further comparison is shown in Fig. 21 inform of bar graphs highlighting the superiority of fuzzy logic model against the mathematical models. Results of the assessment of the state of cellulose paper by the proposed fuzzy logic model were comparable with those of the insulation paper predicted by mathematical models and measured DP values by the utility.

**Table 12:** Degree of Polymerization comparison.

Tx. No.	Utility DP	Chendong [43]	De Pablo [44]	Pahlavanpour [45]	Fuzzy Model [28]	ANFIS [12]	Proposed model	%Error
Tx1	650	603	785	764	643.4	650	648	0.31
Tx2	750	717	798	785	756.2	742	754	0.53
Tx3	840	867	804	796	854.1	745	838	0.24
Tx4	220	244	533	434	172.7	225	185	15.91
Tx5	680	619	787	769	665.7	670	667	1.91
Tx6	780	717	798	785	786.3	786	778	0.26
Tx7	1030	917	805	797	997.5	1018	1002	2.72
Tx8	260	263	559	464	265.5	261	263	1.15
Tx9	600	455	737	693	594.4	554	604	0.67
Tx10	375	299	606k	519	370.3	370	374	0.27
Tx11	1090	1003	806	799	1003.5	1044	1015	6.88
Tx12	470	409	710	654	481.8	463	468	0.43
Tx13	360	345	657	583	351.4	355	360	0.00
Tx14	350	409	710	654	356.7	353	348	0.57
Tx15	500	294	600	512	494.3	497	534	6.8

The model showed greater accuracy and reliability, a DP value of greater than 1000 was obtained for transformers 7 and 11. This DP value signifies a very healthy paper insulation. This result is a true mapping of low concentrations of 2-FAL *(*0.02 ppm*)*, MeOH of 0.02 ppm and also low CO_2_ and CO gas evolving in the insulation at a ratio of 6.52 and 2-FAL of 0.01 ppm, MeOH of 0.01 and an oxide ratio of 3.96 respectively. Similarly, DP value of less than 250 was obtained in transformer 4 indicating the end of life, also a true reflection from the input data of 4.53 ppm for 2-FAL, 2.2 for MeOH and high concentrations of carbon oxides at a ratio of 2.75. Using an old transformer Tx 4, the fuzzy model result reflects accelerated paper aging leading to an end-of-life DP value of l85. The measured utility DP value of 220 also signifies an end-of-life state of the paper insulation. The accelerated degradation of solid insulation is attributed to high furan concentration of 4.53 ppm which is far above threshold value basing on (IEC 61198*)*. In addition, very high concentration of CO_2_ and CO and MeOH, indicates severe degradation of cellulose. To safeguard the transformer operation, exercising of caution in condition monitoring of several factors is recommended. As noted in Ref. [30], if careful consideration of network conditions is taken and especially avoiding mechanical risks, this transformer with such low paper DP value may be kept in service for several more years, possibly, without significantly increasing the risk of failure.

iii. Transformer DOH/DOF Results

Sample test data from different transformers and sources highlighted in Table 8a, Table 8b were used in the validation of the developed DOH/DOF model. The validation was based on a percentage value that was mapped into the linguistic label depending on the status region the transformer falls in (Fig. 1) after inputting the test data of the identified transformers. The DOH, DOF, and incipient fault results for 15 representative transformers whose mapped status outcomes are as given in Table 13. Additionally, Table 13 highlights the corresponding scores for DGAF, OQF, DPF, and MF computed from the model as per the actual values of transformer parameters. From the observed results, it can be noted that a transformer's degree of healthiness or faultiness is not dependent on an individual variable, but a combination of different attributes paints a better picture of what is transpiring in the operation and condition of the transformer.

**Table 13:** Model results.

Tx. NO	FACTOR SCORES				DP VALUE	DOH %	LABEL	DOF %	LABEL	Tx. STATUS	INCIPIENT FAULT			
	DGAF	OQF	DPF	MF							DP1	DP2	CP	MDP
1	27.09	19.37	15.19	8.67	648	84.29	MOH	1.17	LF	MOH	T1	O	T1-O	U1
2	13.16	10.9	12.28	6.39	**754**	**83.95**	**MOH**	**1.17**	**LF**	**MOH**	–	–	–	–
3	19.52	7.28	17.43	3.34	838	84.59	MOH	1.17	LF	MOH	T1	O	T1-O	T1/T2
4	85.49	39.41	27.31	25.95	**185**	**1.94**	**LH**	**99.23**	**EF**	**EF**	T1	C	T1-C	T1/T2
5	21.36	22.26	13.23	10.88	667.0	83.94	MOH	1.18	LF	MOH	S	S	S	U3
6	22.66	25.08	8.47	8.04	778	80.51	MOH	1.17	LF	MOH	T2	C	T2-C	T1/T2
7	9.61	4.60	3.73	4.43	1002	80.83	MOH	1.17	LF	MOH	S	S	S	U5
8	55.09	55.58	23.5	21.67	263	36.18	MH	54.49	F	F	S	S	S	U5
9	19.28	19.35	15.77	14.64	604	80.51	MOH	1.11	LH	MOH	PD	PD	PD	U2
10	17.88	48.27	12.02	15.43	755.6	80.51	MOH	20.36	MF	H	S	S	S	U5
11	5.47	6.17	2.35	1.90	1015	99.25	EH	1.11	LF	EH	–	–	–	–
12	33.15	36.59	14.29	18.25	**468**	**1.95**	**LH**	**1.18**	**LF**	**ROU**	T1	O	T1-O	U1
13	54.68	42.95	17.07	28.29	360	1.95	LH	83.93	MOF	MOF	S	S	S	U5
14	52.76	25.45	16.92	23.57	**348**	**2.25**	**LH**	**20.34**	**MF**	**MF**	S	S	S	U4
15	27.18	23.53	15.15	18.38	534	36.84	MH	1.17	LF	MH	T1	O	T1-O	T1/T2

For illustration purposes, using transformer 2 and 11 from Table 13, although both transformers are not showing signs of transformer faulting based on DGA-MCDP interpretation, their DOH and DOF falls into different regions. If DGA attribute was solely used to valuate DOH/DOF would have led to different outcome which might not be the true representation of the transformer status. Even though, transformer 11 is extremely healthy while transformer 2 is more healthy, the difference is attributed to the fact that for transformer 11, the other factors based on the scores lies within the extreme healthy region and also the paper condition is excellent with the model estimation of 1015 indicating an excellent solid insulation condition while a value of 754 for transformer 2 which is at the boundary to moderate deterioration.

Transformer 4 is classified as extremely faulty with a DOF 99.23 %, it is noted the DP estimated is way below 250 indicating the transformer's severe reduction in its paper insulation mechanical strength and ability to withstand short circuits, indicating that the life of the transformer has reached the end. This is also supported by the fact that the transformer suffers possible carbonization of paper insulation as detected by Duval Pentagon 2 and CDP. Additionally, this transformer suffers thermal faults. Transformer 8 is classified to be in the region of uncertainty (ROU). Both the DOH and DOF are low, least healthy and least faulty respectively as per the fuzzy membership functions. Generally, transformer with values less than 10 % on DOH and DOF is categorized as being in region of uncertainty. A transitional region where the transformer health condition experiences a shift from general healthy to start operating in the faulty region as analogically demonstrated in Fig. 1.

Results from the Pentagons show that the transformer presence of thermal Fault <300 °C, Overheating <250 °C, and a possibility of T1/T2, PD, D2 according to DP1, DP2 and modified Duval Pentagon respectively. Transformer 14 with DOH of 2.25 % and a DOF of 20.34 % is detected to be undergoing stray gassing and PD as per the fault diagnostic utilizing the DGA and the Duval pentagons approach. By subjective reasoning taking into account the two percentages the transformer status is taken as medium faulty. The DP too is low indicating extensive deterioration also to note is that the dissolved gas analysis factor is too high agreeing on the presence of discharges as shown in Table 9.

The degree of healthiness or faultiness assessed by the DOH/DOF fuzzy inference model is a significant characteristic that must be examined consistently so as to determine the asset's overall condition. It may be estimated regularly for critical equipment such as transformers using regularly available data especially from online monitors. This will aid in determining the aging rate of the insulation system and/or discovering faults. As a result, the asset manager will be able to make more informed judgments about resource allocations especially in maintenance and loading strategies. Transformers that exhibit poor health should be given high priority, and vice versa. Similarly, only transformers with a high degree of healthiness should be employed when planning operations to ensure full optimization. For instance, when the DOH of a transformer falls within the region of uncertainty depicted in Fig. 1, actions like overloading that transformer during excessive loads should be inadmissible.

## 5 Conclusion

This paper introduces a methodology of synthesizing the DOH and DOF of mineral oil-filled transformers in addition to exploration of incipient fault diagnostics based on group factor score calculation and fuzzy logic inference approach. The establishment of the DOH/DOF model was based on the fuzzy logic inference tool centered upon the computation of cumulative grouped factors that signifies the transformer condition established on the Dissolved Aas analysis Factor (DGAF), oil quality analysis factor (OQF), degree of polymerization factor (DPF), and transformer maintenance data factor (MF). Since DP value mirrors the condition of the solid insulation of the transformer which is a crucial factor in determining the DOH/DOF, a non-intrusive method for DP estimation was established in this paper. The DP value was established from a modified fuzzy logic-based model using Furans (2-FAL), carbon oxide ratio, and methanol as the inputs. Comparing the modified DP model's outcome with the power utility and other models DP values for diverse transformers, it was observed that the proposed model output was more comparable with utility's and the ANFIS results and better than the traditional mathematical models.

The overall resultant of the DOH/DOF valuation was governed by all factors taken into account collectively rather than any solely pointed specific individual parameter. From the observed results, transformers with label MH between 30 % < DOH <75 % or MOH of DOH of at least 75 % can be subjected to time limit overloading as per manufacturers' guiding principle. However, the one with DOH <30 % should not be subjected to stressful conditions as they are prone to failure. Equally, transformers whole value for degree of faultiness is above 50 % must be continuously be monitored and should operate under low loading conditions. However, those transformers that falls in the EF of DOF of at least 90 % must be subjected to urgent planned maintenance or even shut down depending on the loading and line of operation. Continuous use of these transformers can lead to disastrous failure. The evaluation's findings have unequivocally demonstrated DOH/DOH's importance, particularly in applications like scheduling for operations, maintenance, and replacements of in-service power transformers. However, it can be noted that if more dependable findings of equipment healthiness are to be achieved, both the magnitude and the incremental rate of parameter change be taken into consideration.

Additionally, the fault diagnosis results highlighted that the modified combined Duval Pentagon (MCDP) strategy is more robust as compared to the individual pentagons basing of the six IEC diagnosed faults. Notably from a sample size of sixty-five (65) physically inspected cases of pre-known incipient faults, it exhibited a total diagnostic accuracy of 95.4 %, which is a higher value when compared to total accuracy of 81.4 % of the conventional Combined Duval Pentagon (CDP). The CDP had a low detection accuracy of thermal faults of below 700 °C (T1/T2) at 40 % as most of the point were detected as thermal faults of high temperature >700 °C (T3). MCDP managed a 100 % detection accuracy in partial discharges (PD) and discharges of low energy (D1). Other than its potential high fault detection accuracy, the MCDP has the ability to detect multiple faults which are simultaneously building inside the transformer. However, this school of thought of fault diagnosis based on DGA still needs to be explored further as different approaches leads to different results. Further research will focus on cascading the MCDP with artificial intelligence techniques in transformer fault diagnosis. Power transformer dependability and residual life can both be improved by continuously having the knowledge of its degree of healthiness or faultiness.

## CRediT authorship contribution statement

**J.M. Makacha:** Writing – original draft, Visualization, Validation, Software, Resources, Methodology, Formal analysis, Data curation, Conceptualization. **Edwell T. Mharakurwa:** Writing – review & editing, Visualization, Validation, Supervision, Software, Resources, Methodology, Data curation, Conceptualization. **L.O. Mogaka:** Writing – review & editing, Visualization, Validation, Supervision, Software, Methodology, Data curation.

## Ethical statement

This study does not involve any human or animal subjects, and it is in accordance with research ethical standards.

## Data availability

Selected test data used in model evaluation has been provided in this article.

## Declaration of competing interest

The authors declare that they have no known competing financial interests or personal relationships that could have appeared to influence the work reported in this paper.
